# Trigger finger in children with hurler syndrome – distribution pattern and treatment options

**DOI:** 10.3205/iprs000154

**Published:** 2021-05-05

**Authors:** Andreas Jokuszies, Lorenz Grigull, Tobias Mett, Khaled Dastagir, Alperen Bingoel, Peter M. Vogt

**Affiliations:** 1 Department of Plastic, Aesthetic, Hand and Reconstructive Surgery, Hannover Medical School, Hannover, Germany; 2 Department of Pediatric Hematology and Oncology, Hannover Medical School, Hannover, Germany

**Keywords:** disorder, hurler, lysosomal, mucopolysaccharide, trigger finger

## Abstract

**Introduction:** Mucopolysaccharidosis is a rare and congenital autosomal recessive lysosomal storage disorder of glycosaminoglycans. An enzyme defect leads to cell, tissue and organ dysfunction. Carpal tunnel syndrome and trigger finger are the results of mucopolysaccharid deposition.

**Material and methods:** We are treating 6 patients with mucopolysaccharide associated trigger fingers in an interdisciplinary setting with the department of pediatric hematology and oncology at Hannover Medical School, where each patient is examined inter alia for symptoms of trigger finger annually.

Besides an interview of the parents about abnormalities with regard to hand function, pain and/or neurologic symptoms the children are examined by palpation and by assessment of the active and passive range of finger motion.

In the case of finger locking due to an impaired excursion of the flexor tendons in the A2 and A3 pulley region, we performed a trap-door incision technique for A2 pulley widening and a simple release of the A3 pulley.

**Results:** In 6 patients 43 fingers were affected. The average age was 10 years. Pulley thickening was palpated in 19 fingers of to the left hand and 24 fingers of the right hand. In 7 fingers the A1 pulley was affected, in 28 fingers the A2 pulley and in 25 fingers the A3 pulley. The A4 and A5 pulley were not affected in any case. Trigger symptoms were seen in 13 fingers. Five of the 6 children were given an operation indication. In these cases we performed carpal tunnel release, release of Loge de Guyon, and trigger finger release, either in combination or alone. In all cases the procedure led to pain relief and functional improvement.

**Conclusion:** The treatment of trigger fingers in children with mucopolysaccharidosis as a rare disease is challenging with regard to diagnostics and indication.

The main treatment goal is pain relief and improvement of hand function.

## Zusammenfassung

**Einleitung:** Die Mukopolysaccharidose ist eine seltene und angeborene, autosomal rezessive und lysosomale Speicherkrankheit von Glykosaminoglykanen. Ein Enzymdefekt führt zu Zell-, Gewebe- und Organfunktionsstörungen. Das Karpaltunnelsyndrom und der schnappende Finger sind die Folge der Mukopolysaccharidablagerungen.

**Material und Methode:** Wir behandeln zurzeit 6 Patienten mit Mucopolysaccharid-assoziiertem Schnappfinger in einem interdisziplinären Setting zusammen mit der Abteilung für pädiatrische Hämatologie und Onkologie an der Medizinischen Hochschule Hannover. Jeder Patient wird im Rahmen der jährlichen Kontrolluntersuchungen unter anderem im Hinblick auf die Symptome eines schnappenden Fingers hin untersucht.

Neben einer detaillierten Befragung der Eltern zu bestehenden Funktionsstörungen der Hände ihrer Kinder, begleitenden Schmerzen und/ oder neurologischen Symptomen werden die Kinder eingehend klinisch durch Palpation und mit Bestimmung der aktiven und passiven Beweglichkeit der Finger untersucht.

In den Fällen mit auslösbarem Schnappphänomen der betroffenen Finger und Blockade auf Höhe der A2- und A3-Ringbänder wurde die Indikation zur A2-Ringbanderweiterungsplastik und A3-Ringbandspaltung gestellt.

**Ergebnisse:** Bei den 6 untersuchten Patienten waren insgesamt 43 Finger betroffen. Das durchschnittliche Alter der Kinder lag bei 10 Jahren. Eine Verdickung der Ringbänder fand sich bei 19 Fingern der linken Hand und bei 24 Fingern der rechten Hand. Bei 7 Fingern war das A1-Ringband betroffen, bei 28 Fingern das A2-Ringband und bei 25 Fingern das A3-Ringband. Das A4- und A5-Ringband war bei keinem der untersuchten Patienten betroffen. Ein Schnappphänomen beobachteten wir bei insgesamt 13 Fingern. Bei 5 der 6 Kinder stellten wir eine Operationsindikation. In diesen Fällen erfolgten, entweder kombiniert oder isoliert, eine Karpaldachspaltung, eine Spaltung der Loge de Guyon und eine A2-Ringbanderweiterungsplastik und A3-Ringbandspaltung. In allen Fällen führte der Eingriff zu einer Schmerzfreiheit und Funktionsverbesserung der Hände durch Ausbleiben der Fingerblockade.

**Fazit:** Die Behandlung des schnappenden Fingers bei Hurler-Kindern betrifft v.a. das A2- und A3-Ringband und erfordert eine genaue Diagnostik und Indikationsstellung. Schmerzfreiheit und die Funktionsverbesserung der Hände sind das primäre Ziel.

## Introduction

The Hurler syndrome (mucopolysaccharidosis-1H) is a very rare inherited disorder and one of seven types of mucopolysaccharide (MPS) storage diseases [1], [2].

In the hand, its clinical characteristics like joint stiffness, trigger finger and carpal tunnel syndrome are the result of an excessive glycosaminoglycan deposition in the connective tissue and tendon sheets.

Whereas other authors have described their experience in the diagnosis and treatment of Hurler children suffering from clinical signs of carpal tunnel syndrome with stenosis of the A1 pulley, in our study, we observed the A2 and A3 pulleys being predominantly affected and therefore focused on their distribution pattern and our choice of treatment [3], [4], [5].

As a simple release of the A2 pulley would lead to a bow stringing we present our trap-door incision technique for pulley widening. The A3 pulley was released by simple incision.

## Material and methods

Since 2006 we are treating 11 children with Hurler syndrome in an interdisciplinary setting in our institution. Six of these 11 children are under continuous observation, whereas the other 5 present at irregular intervals for logistic and/or personal reasons.

Two of them are treated by other clinics in the Netherlands and Germany.

The children are annually referred to us by our colleagues from the department of pediatric oncology and hematology for follow-up of hand associated Hurler symptoms.

The children underwent stem cell transplantation and undergo a frequent and comprehensive diagnostic procedure program in which they are presented to pediatric ophthalmologists, neurologists, cardiologists, otorhinolaryngologists, orthopedics and hand surgeons due to the affected organ (every 12 months).

During their consultation at our department, the children undergo a detailed investigation of their hand function with focus on carpal tunnel and trigger digit symptoms. This investigation is complemented by a detailed interrogation of the parents regarding their perception of their children’s hand skills, neurologic symptoms, handedness, and potential nail biting.

**Figure 1:**
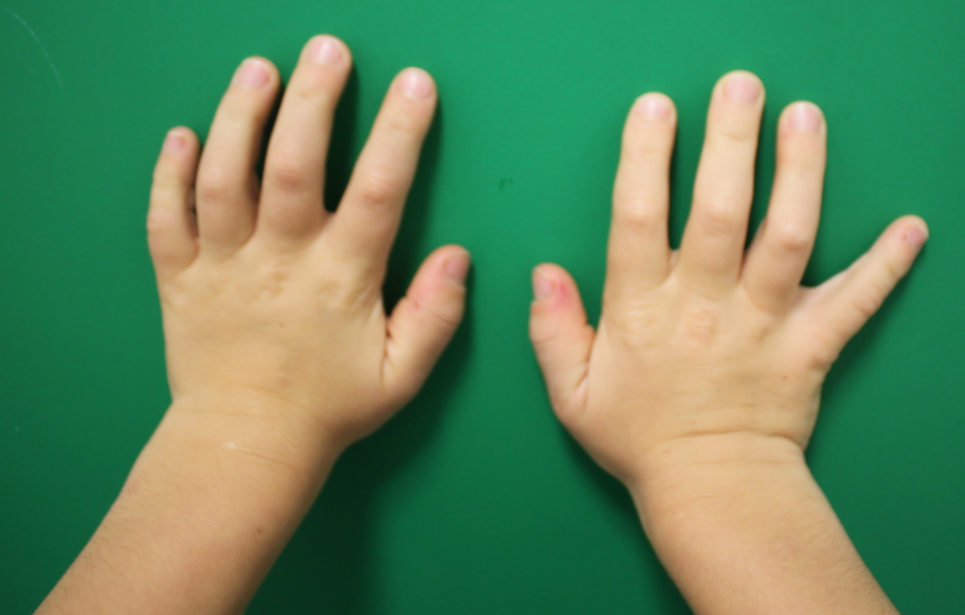
Typical Hurler hands with wide and broad fingers and limited extension

The typical appearance of Hurler hands are wide and short fingers with combined stiffness of the metacarpophalangeal and interphalangeal joints which leads to limited extension (Figure 1 (Fig. 1)) [6].

**Figure 2:**
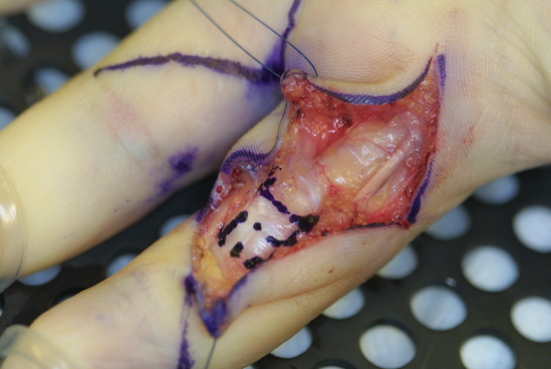
Trap-door incision of the A2 pulley

In those children whose hand function and skills were obviously impaired due to pain, neurologic symptoms, and locking of finger excursion, we performed surgery by carpal tunnel release, release of Loge de Guyon, and trigger finger release. As the A2 pulley prevents the flexor tendons from bow stringing, it was widened by trap-door incision whereas a simple release by longitudinal incision was performed in the A3 pulley (Figure 2 (Fig. 2)). As the A1 pulley was not thickened and painful under palpation, we saw no indication for an A1 pulley release.

After being discharged on the second postoperative day, further treatment entails a bandage for the first 7 days followed by occupational therapy and physiotherapy with focus on active and passive finger mobilization while removal of the absorbable sutures is not necessary.

## Results

In our study population of 6 children, 43 fingers were involved in total. The mean age was 12 years. Considering the site, we observed 19 fingers with thickened pulleys in the left hand and 24 affected fingers in the right hand.

**Figure 3:**
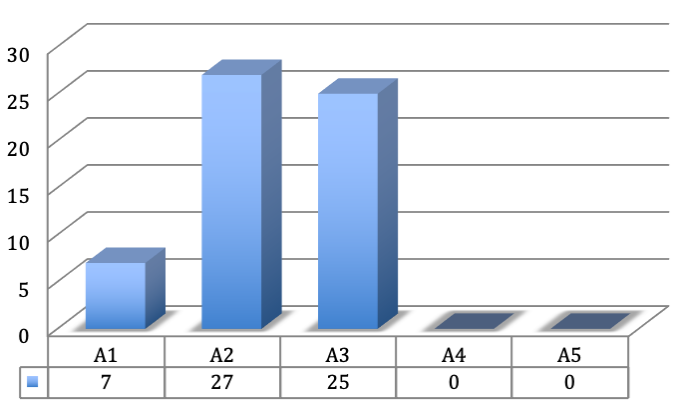
Frequency of the affected pulleys

In 7 fingers the A1 pulley was affected, in 27 fingers the A2 pulley and in 25 fingers the A3 pulley (Figure 3 (Fig. 3)).

The A4 and A5 pulley were not affected in any case. Trigger symptoms were seen in 13 fingers. Five of the 6 children were given an operation indication because of painful and intermittent flexion contracture of the affected finger and resulting impairment of hand function.

In these children we performed carpal tunnel release, release of Loge de Guyon, and trigger finger release by trap-door incision of the A2 pulley and A3 pulley release through longitudinal incision, either in combination or alone. In all cases, the procedure led to pain relief and functional improvement as reported by the parents and observed by clinical investigation.

In all cases, triggering of the affected finger was absent and could not be provoked by active and passive finger excursion as preoperatively. In no case, bow stringing of the flexor tendons occurred and no recurrence was observed so far.

With focus on the distribution of the affected pulleys and according to the digits of both hands it is obvious that in most of the cases the pulleys A2 and A3 were affected, which is atypical for trigger fingers. The A1 pulley was affected only in 5 cases, in which both thumbs were affected in two children and the left thumb in a further child.

**Figure 4:**
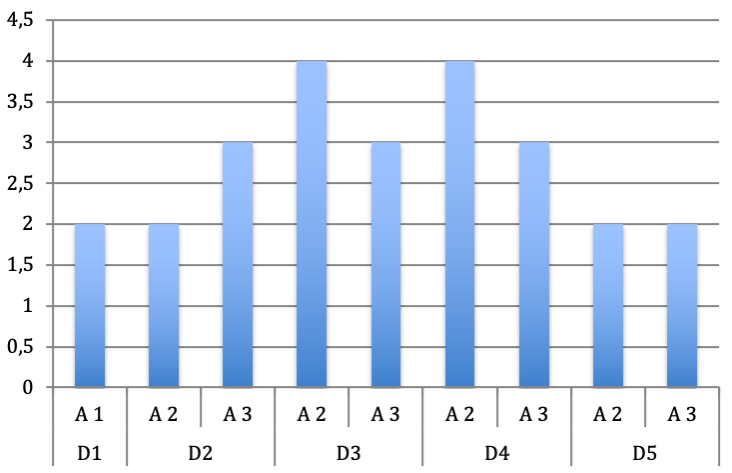
Distribution of the affected pulley according to the digits of the right hand

**Figure 5:**
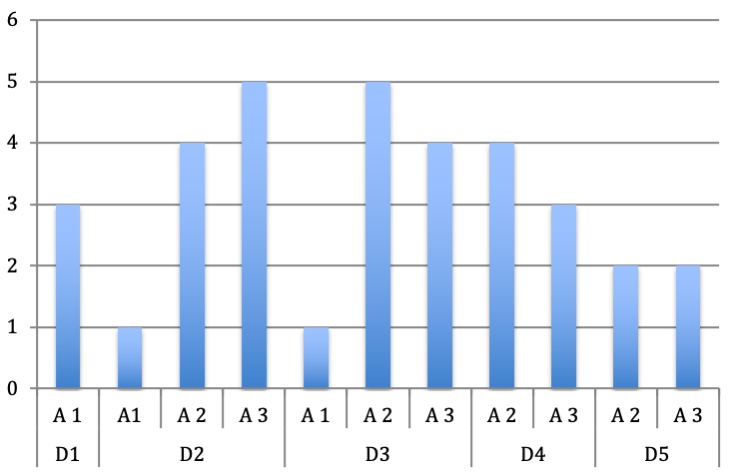
Distribution of the affected pulley according to the digits of the left hand

Only in one of the six children the A1 pulley was affected in the index and middle finger of the left hand (Figure 4 (Fig. 4) and Figure 5 (Fig. 5)).

**Figure 6:**
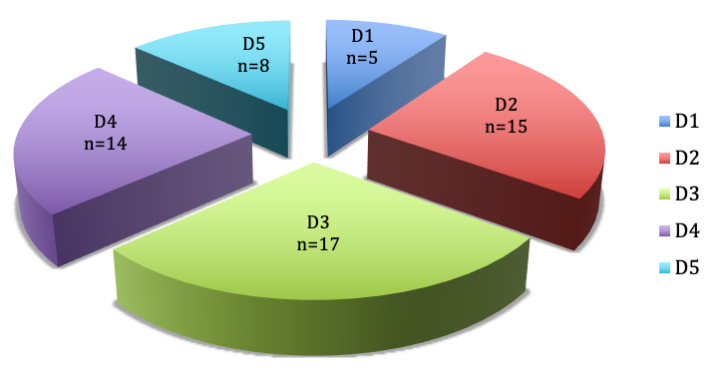
Distribution of the affected pulley according to the digits

Considering the affected finger, we observed a dominant distribution towards the index, middle and ring finger (Figure 6 (Fig. 6)). 

**Figure 7:**
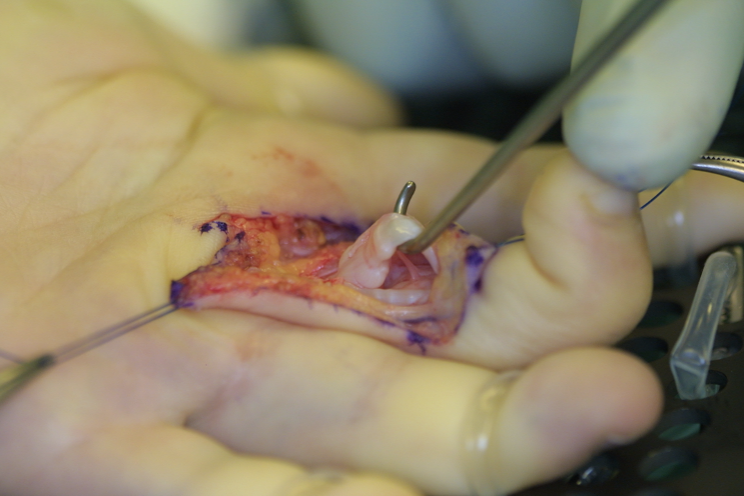
‘Pearl of string’ like appearance of the flexor tendon

Intraoperatively, we observed a “pearl of string” like appearance of the flexor tendons due to an excessive mucopolysaccharide deposition resulting in a sliding barrier for the flexor tendons (Figure 7 (Fig. 7)).

The histologic specimen of the tendon sheets revealed connective tissue containing fibrosis and macrophages typical for mucopolysaccharidoses whereas inflammatory signs were not present.

## Discussion

As Hurler syndrome is a very rare disease with different organ systems being affected, children are treated in specialized centers in an interdisciplinary setting [3].

This means that we are able to follow up a large patient collective over a long period of time. 

Trigger fingers are very common in patients with Hurler syndrome but very little is known about their distribution pattern and the treatment options.

Whereas usually the A1 pulley is affected in trigger finger in adults and children with pollex flexus congenitus, we observed predominantly the A2 and A3 pulley being affected in Hurler children [7], [8]. 

This is in accordance with an observation made by Wyffels et al. who observed palpable nodules along flexor tendons particular distal to the A2 pulleys in all fingers of a twenty-three-year-old patient in whom they performed A1 and A3 pulley release, tenosynovial resection, and partial flexor digitorum superficialis slip resection to allow better tendon excursion [9].

As we interpret their approach, they only performed tenosynovectomy in the carpal tunnel region and in accordance with our approach widened the space under the annular pulleys. The difference between the two approaches is the way of widening the space under the annular pulleys which we decided to perform by a combination of pulley widening and release leaving the tendon undisturbed to prevent possible and subsequent rupture due to weakening if resecting the deposits.

Kim et al. showed by high resolution ultrasonography that the severity of trigger fingers is not only associated with a thickened A1 pulley but also with a thickening and stiffness of the A2 pulley and the cross-sectional area of the flexor tendon under the A2 pulley, which confirms our observation and results [10]. 

Whereas the pathophysiology of trigger finger is still controversially discussed, there is no doubt that it is a result of a mismatch between the volume of the flexor tendon sheet and the tendon [11].

The typical examination findings are a knotlike thickening of the affected pulleys by palpation. Active and passive finger flexion and extension produces pain and locking.

In our cases, the tendons showed a “pearl of string” like appearance intraoperatively due to an excessive mucopolysaccharide deposition resulting in a sliding barrier for the flexor tendons.

In accordance with Drossos et al., the histologic specimen of the tendon sheets revealed connective tissue containing fibrosis and macrophages typical for mucopolysaccharidoses and were not related to inflammation [12]. 

The open approach with success rates of 99% is still the “gold standard” in which a complete trigger finger release is ensured by a longitudinal excision [13], [14].

Additionally, it helps to identify further pathologies like rare cases of atraumatic tendon ruptures or structural changes as in our presented cases [14].

Tendon ruptures or weakness of the tendons due to mechanical stress could not be observed in our study. To avoid weakening of the tendons by tangential flattening of the “pearl string” like mucopolysaccharide depositions, we instead performed a combined trap door widening of the A2 pulley and release of the A3 pulley with very good results. So far all the children are free of symptoms.

## Conclusion

Trigger finger is a common pathology in Hurler syndrome and one should beware of involvement of the A2 and A3 pulleys in treatment planning. To prevent bow stringing and to ensure a free gliding of the flexor tendons, we recommend a combined A2 trap-door incision and A3 release for pulley widening.

Long-term monitoring has to prove whether our approach is worthwhile in prevention of trigger symptoms in the aged. 

## Notes

### Competing interests

The authors declare that they have no competing interests.

No benefits in any form have been received or will be received related directly or indirectly to the subject of this article.

### Informed consent

The patients have given informed consent to publication.
